# SIRT1 Activity in Peripheral Blood Mononuclear Cells Correlates with Altered Lung Function in Patients with Chronic Obstructive Pulmonary Disease

**DOI:** 10.1155/2018/9391261

**Published:** 2018-05-09

**Authors:** Valeria Conti, Graziamaria Corbi, Valentina Manzo, Paola Malangone, Carolina Vitale, Angelantonio Maglio, Roberta Cotugno, Damiano Capaccio, Luigi Marino, Carmine Selleri, Cristiana Stellato, Amelia Filippelli, Alessandro Vatrella

**Affiliations:** ^1^Department of Medicine, Surgery and Dentistry, University of Salerno, Via S. Allende, 84081 Baronissi, Italy; ^2^Department of Medicine and Health Sciences, University of Molise, Via Francesco De Sanctis, 86100 Campobasso, Italy; ^3^Department of Pharmacy, University of Salerno, Via Giovanni Paolo II, 84084 Fisciano, Italy; ^4^Maria Ss. Addolorata Hospital, Piazza Scuola Medica Salernitana, 84025 Eboli, Italy

## Abstract

**Background:**

Oxidative stress is a recognized pathogenic mechanism in chronic obstructive pulmonary disease (COPD). Expression of the NAD^+^-dependent deacetylase Sirtuin 1 (SIRT1), an antiaging molecule with a key role in oxidative stress response, has been described as decreased in the lung of COPD patients. No studies so far investigated whether systemic SIRT1 activity was associated to decreased lung function in this disease.

**Methods:**

We measured SIRT1 protein expression and activity in peripheral blood mononuclear cells (PBMCs) and total oxidative status (TOS), total antioxidant capacity (TEAC), and oxidative stress index (TOS/TEAC) in the plasma of 25 COPD patients, 20 healthy nonsmokers (HnS), and 20 healthy smokers (HS).

**Results:**

The activity of SIRT1 was significantly lower in COPD patients compared to both control groups while protein expression decreased progressively (HnS > HS > COPD). TOS levels were significantly lower in HnS than in smoke-associated subjects (COPD and HS), while TEAC levels were progressively lower according (HnS > HS > COPD). In COPD patients, SIRT1 activity, but not protein levels, correlated significantly with both lung function parameters (FEV1/FVC and FEV1) and TEAC.

**Conclusions:**

These findings suggest loss of SIRT1-driven antioxidant activity as relevant in COPD pathogenesis and identify SIRT1 activity as a potential convenient biomarker for identification of mild/moderate, stable COPD.

## 1. Introduction

Chronic obstructive pulmonary disease (COPD) is a common, preventable, and treatable disease that is characterized by persistent respiratory symptoms and airflow limitation that is due to airway and/or alveolar abnormalities usually caused by significant exposure to noxious particles or gases [1]. Although current therapies are able to attenuate symptoms and prevent exacerbations, they do not influence the progression of the disease [2, 3].

In COPD genetic, environmental and epigenetic factors cooperate to generate chronic inflammation, leading to emphysema and irreversible airway remodeling [4, 5]. Irrespective of different clinical phenotypes, the progressive decline in lung function is a common defining characteristic [5, 6]. It has been suggested that COPD is the phenotypic expression of an accelerated aging process of the lung caused, at least in part, by decreased efficiency of several antiaging molecules, including those regulating cellular redox state and oxidative stress response [7, 8].

Oxidative stress is one of the driving molecular mechanisms in COPD. Cigarette smoke (CS) represents the major environmental risk factor for COPD, containing more than 10^14^ oxidants/radical oxygen species (ROS) per puff. In COPD patients, it is very frequent to recognize an impairment of the endogenous antioxidant system with a consequent increase of ROS and inflammatory mediators that in turn accentuate ROS production, creating a vicious cycle [4, 9]. Therefore, chronic cigarette smoke critically contributes to an oxidant/antioxidant imbalance in favor of an increased burden of ROS and oxidants [4, 10].

Clinical and experimental evidence indicates that inflammation and oxidative stress occurring in the lungs during COPD are coupled with systemic inflammation, correlating with the disease [11]. In fact, overexpression of prooxidant and proinflammatory molecules found in both lung and peripheral blood compartments has been correlated with lung function parameters indicating airway obstruction and its severity, such as forced expiratory volume in one second (FEV1)/forced vital capacity (FVC) and the FEV1, respectively [12–14]. Sirtuin1 (SIRT1) is the best-characterized member of the protein family of sirtuins, a group of seven deacetylases that target histone and nonhistone proteins and require NAD(+) as enzymatic cofactor. SIRT1 is considered as an antiaging molecule that modulates the response to both oxidative and inflammatory stressors through deacetylation of several proteins such as Forkhead box O3 A (Foxo3a) and nuclear factor kappa b (Nf-kB) [15]. SIRT1 overexpression and the use of SIRT1 activators are able to dramatically decrease oxidative stress induced by CS [16]. Because of its crucial role in modulating the oxidative stress response, SIRT1 dysfunction has been implicated in aging and several aged-associated diseases including COPD [7, 17–20]. In particular, decreased levels of SIRT1 expression have been found in the peripheral lung and in serum of COPD patients compared to healthy controls [21–23]. However, while changes in the expression levels of SIRT1 have been monitored, no data indicate so far whether SIRT1 activity is also affected in COPD patients and whether it would correlate with lung function.

The aim of this study is to analyze SIRT1 function in the peripheral blood mononuclear cells (PBMCs) of patients with COPD compared to nonsmokers and smokers with normal lung function, by quantifying its activity along with protein expression and investigating the relationship between SIRT1 activity and markers of oxidative stress as well as with parameters of lung function in the three groups.

## 2. Material and Methods

### 2.1. Participants

An observational study was conducted on patients with stable COPD (named COPD from here on), consecutively admitted at the Pneumology Unit of the University Hospital of Salerno, Italy. We enrolled volunteers as controls, either smokers (Healthy Smokers, HS) or never-smokers (Healthy non-Smokers, HnS), considered as Healthy on the basis of their normal lung function. The recruitment record included demographic variables and medical history. Smoking history was evaluated by pack years of cigarette consumption. Exclusion criteria were COPD exacerbations and respiratory tract infection that had required antibiotics within the last 3 months prior to enrollment; supplementation with antioxidants; and administration of any of the following drugs: allopurinol, N-acetylcysteine, or oral corticosteroids over the last six months. Patients who had a history of respiratory diseases, cancer, myocardial infarction, angina, heart failure, stroke, and disorders of the central nervous system were also excluded.

All participants provided their written informed consent. This observational study was approved by the local ethics committee, in accordance with the Declaration of Helsinki and its amendments and was performed without interfering with normal clinical practice.

### 2.2. Assessment of Pulmonary Function

All subjects underwent pulmonary function testing (PFT) by using a standard spirometer (Vmax® Encore PFT System-CareFusion-BD, USA). All measurements were performed according to the standards established by the American Thoracic Society.

According to the GOLD criteria [1], the diagnosis of COPD was made when a value below 70% in the ratio between the forced expiratory volume in one second (FEV1) and the forced vital capacity (FVC) was obtained.

### 2.3. Blood Sampling

Blood samples were collected in fasting condition in BD Vacutainer® containing sodium heparin (BD, USA). The separation of plasma and peripheral blood mononuclear cells (PBMCs) was obtained by Ficoll density gradient centrifuged at 3000 rcf spin for 30 minutes at room temperature. Aliquots of plasma and PBMCs were frozen at −80°C until further analysis.

### 2.4. SIRT1 Protein Expression and Activity

SIRT1 protein expression and activity were evaluated in nuclear extracts (10 *μ*l) isolated by PBMCs using a nuclear extraction kit (EpiGentek Group Inc.).

SIRT1 expression was measured by enzyme-linked immunosorbent assay (SIRT1 ELISA Kit, Elabscience), following the manufacturer's instructions. SIRT1 activity was determined using a SIRT1/Sir2 Deacetylase Fluorometric Assay (CycLex, Ina, Nagano, Japan), following the manufacturer's instructions. Values were reported as relative fluorescence/*μ*g of protein (AU). All data are expressed as mean ± SD of three independent experiments.

### 2.5. Oxidative Stress Markers

Total oxidant status (TOS) and Trolox equivalent antioxidant capacity (TEAC) were measured in plasma isolated from HnS, HS, and COPD. The oxidative stress index (OSI) was calculated as TOS/TEAC. Data are the means ± SD of three independent experiments.

### 2.6. TOS Assay

To evaluate TOS, the content of peroxides in plasma samples was measured by the oxidation of ferrous ions in the presence of xylenol orange in acidic environment [24]. Briefly, ferrous–xylenol orange (FOX) reagent was prepared in 25 mM sulfuric acid (pH 1.75) by adding 250 *μ*M ferrous sulfate, 150 *μ*M xylenol orange, and 100 mM sorbitol.

96-well plates were filled with 237.5 *μ*l of FOX reagent and 12.5 *μ*l of sample. After 10 min incubation at room temperature under gentle mixing, the absorbance was measured at 560 nm (main wavelength) and at 800 nm (secondary wavelength). The concentration of peroxides in the samples was calculated using a standard curve of H2O2. In each experiment, two concentrations of H2O2 were tested in the presence of catalase. The lack of any increase of absorbance confirmed the specificity of the FOX reagent.

### 2.7. TEAC Assay

TEAC was quantified by the 2,2′-azino-bis (3-ethylbenzothiazoline-6-sulphonic acid) (ABTS) assay [25] in which stable ABTS+ radical was generated by mixing 5.8 mM ABTS with 2 mM ammonium persulfate (NH4)2S2O8 in 100 mM PBS (pH 7.4) and incubating the mixture in the dark at room temperature overnight. Then, the ABTS+ radical stock solution was diluted with PBS to obtain an absorbance of 0.75 O.D. at 734 nm (working solution). The spectrum of the working solution (400–800 nm) was also analyzed to check its purity.

The assay was performed in 96-well plates by mixing 10 *μ*l of plasma samples, diluted 1 : 5 with 240 *μ*l of the ABTS+ working solution immediately before using. The absorbance of samples was measured exactly after 2 min after mixing using a microplate reader (Thermo Scientific). A blank containing PBS instead of the plasma was included in the assay. We chose to measure the decrease of absorbance at 2 min because of the presence in human plasma not only of “fast-reacting” antioxidants, for example, vitamin E and C, but also of “late-reacting” antioxidants, such as tyrosine residues of plasma proteins [26, 27]. Due to the short time passing between filling of the wells and absorbance measurements, we analyzed only two plasma samples, in duplicate, at a time. The concentration of “fast-reacting” antioxidants was measured by using a standard curve of Trolox, a vitamin E analogue, and data are expressed as *μ*M Trolox equivalents.

### 2.8. Statistical Analysis

Data were analyzed using the SPSS (v 23.0) software package (SPSS Inc., Chicago, IL, USA). The Shapiro-Wilk Test was used to assess the normal distribution of data. Differences between multiple groups were evaluated by analysis of variance (ANOVA) with the Bonferroni post hoc test and are presented as mean ± SD. The *χ*^2^ test was used to compare categorical variables. A multiple linear or logistic regression analysis was used to investigate the relationship between variables when appropriate. In order to explore correlation between variables, Spearman's correlation (r) was used. The statistical significance was established at *p* < 0.05.

## 3. Results

### 3.1. Participants

A total of 25 COPD patients (COPD) and 40 age-matched individuals with normal lung function (20 healthy smokers (HS) and 20 healthy nonsmokers (HnS)) were included in the study. Main characteristics of all participants are listed in Table 1.

The COPD patients were in stable condition, 17 (68%) with GOLD2 and 8 (32%) with GOLD3. Six patients were long-acting muscarinic agonist (LAMA) users, 8 used LAMA plus long-acting beta agonist (LABA), 3 LABA plus inhaled corticosteroids (ICS), and 8 LAMA + ICS + LABA.

#### 3.1.1. SIRT1 Activity Decreased in PBMCs of COPD Patients Not in Control Groups Regardless of Their Smoking Status

**Figure 1:**
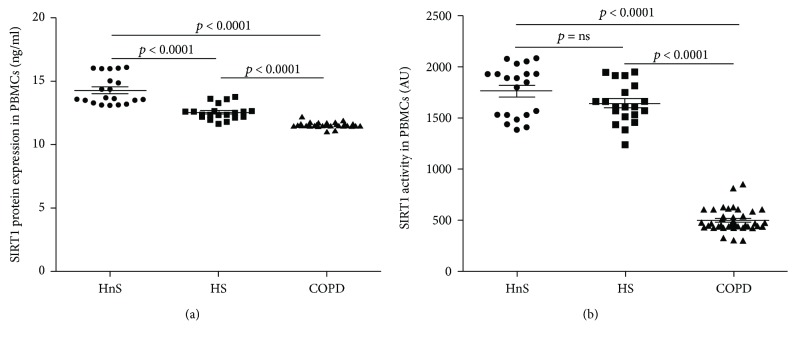
SIRT1 protein expression (a) and activity (b) in peripheral blood mononuclear cells (PBMCs). Data are expressed as mean ± SD. Sirtuin 1 (SIRT1) expression and activity were determined in the nuclei extracted from PBMCs of healthy nonsmokers (HnS), healthy smokers (HS), and COPD patients (COPD), respectively indicated with black circle, black square, and black triangle.

The levels of both SIRT1 expression and activity, evaluated in the PBMCs nuclei, were the lowest in the COPD patients but with different patterns (Figures 1(a) and 1(b)).

Protein expression of SIRT1 decreased in the control groups according to smoking status with the HS group showing a lower value than the HnS, while it was significantly lower in COPD compared to both control groups. Interestingly, SIRT1 activity was uniquely decreased in the COPD compared to both control groups, remaining instead comparable between the HnS and HS.

#### 3.1.2. Oxidative Status Index and TEAC in Peripheral Blood Separate COPD Patients from Smoker and Nonsmoker Controls

Given that SIRT1 is a master regulator of cellular oxidative status and oxidative stress response, we compared plasmatic levels of total oxidative status (TOS), Trolox equivalent antioxidant capacity (TEAC), and oxidative stress index (OSI, TOS/TEAC) among the three experimental groups.

**Figure 2:**
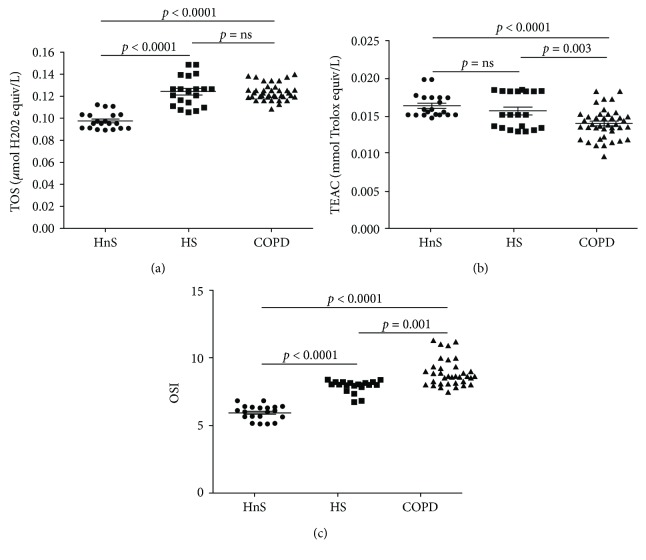
Total oxidative status (TOS) (a), Trolox equivalent antioxidant capacity (TEAC) (b) and oxidative stress index (TOS/TEAC, OSI) (c) in plasma. Data are expressed as mean ± SD. TOS, TEAC, and OSI were determined in the plasma of healthy nonsmokers (HnS), healthy smokers (HS), and COPD patients (COPD), respectively indicated with black circle, black square, and black triangle.

TOS was similar between the COPD and HS groups and significantly higher compared to HnS, indicating similar oxidative burden between the groups exposed to chronic smoke (Figure 2(a)).

Notably, TEAC, which indicate specifically the antioxidant capacity, was significantly lower in the COPD compared to HnS and HS, which instead displayed comparable values (Figure 2(b)).

As expected, OSI (TOS/TEAC) was higher in COPD compared to both control groups as well as in HS versus HnS (Figure 2(c)).

#### 3.1.3. SIRT1 Activity, Not Protein Levels, Correlates with Pulmonary Function and Total Antioxidant Capacity in COPD Patients

**Figure 3:**
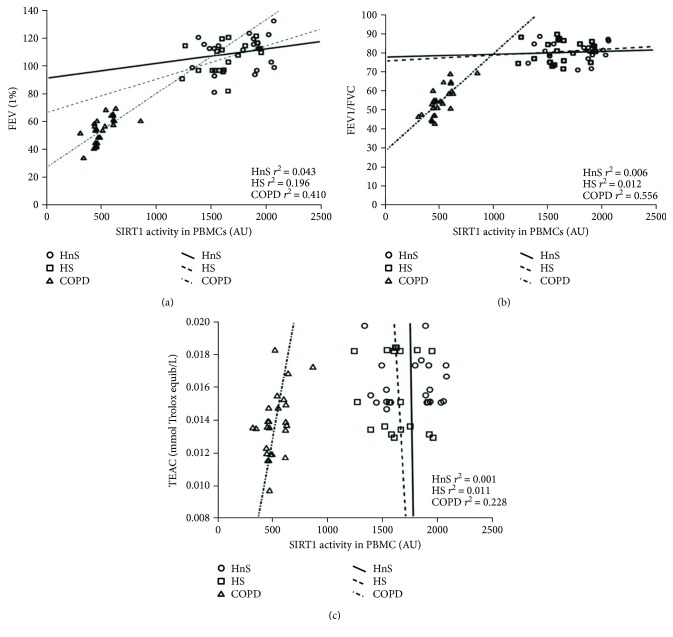
Linear regression analysis between Sirtuin 1 (SIRT1) activity in peripheral blood mononuclear cells (PBMCs) and forced expiratory volume in 1 second (FEV1) values (a); between SIRT1 activity in peripheral blood mononuclear cells (PBMCs) and forced expiratory volume in 1 second/forced vital capacity (FEV1/FVC) ratio values (b); and between SIRT1 activity in peripheral blood mononuclear cells (PBMCs) and serum TEAC (c). SIRT1 activity was determined in the nuclei extracted from PBMCs of healthy nonsmokers (HnS), healthy smokers (HS), and COPD patients (COPD). The multivariate analysis was also adjusted for age, gender, BMI, smoking pack/years, comorbidity, and drugs.

We then compared SIRT1 activity and protein expression levels with corresponding lung function parameters of the three groups. After correction for confounding factors (age, gender, BMI, smoking pack years, comorbidity, and drugs) for COPD patients, we found a statistically significant correlation by linear regression analysis between the SIRT1 activity measured in PBMCs and both FEV1 and FEV1/FVC (*p* < 0.0001, *r*^2^ = 0.848 and *p* < 0.0001, *r*^2^ = 0.780, resp.) (Figures 3(a) and 3(b)).

Such strong correlation (*p* < 0.0001, *r*^2^ = 0.556 and *p* = 0.001, *r*^2^ = 0.410, resp.) was not present for any of the control groups (Figures 3(a) and 3(b)). Moreover, a significant correlation by linear regression analysis was found exclusively in the COPD patients between SIRT1 activity and the TEAC levels (*p* = 0.016, *r*^2^ = 0.228) (Figure 3(c)).

Strikingly, no correlation between SIRT1 expression, lung function parameters, and TEAC levels was found (FEV1: *p* = 0.114, *r*^2^ = 0.103; FEV1/FVC: *p* = 0.513, *r*^2^ = 0.026; TEAC: *p* = 0.866, *r*^2^ = 0.006) in COPD patients.

## 4. Discussion

Our study shows for the first time that levels of SIRT1 activity are decreased in PBMCs of patients with COPD, while remaining comparable between nonsmoking and smoking control groups; instead, SIRT1 protein expression levels were not discriminative for diseased subjects, as they were already significantly decreased in smoker versus nonsmoker controls and becoming further reduced in COPD samples. The latter data are in accordance with previous studies showing decreased protein expression levels of SIRT1 in the peripheral lung and serum of COPD patients [16, 21–23]. Moreover, we found that SIRT1 activity levels also uniquely correlated with airway obstruction and its severity in COPD after correction for confounding factors such as age, sex, comorbidities, and pack/years, while no correlation between SIRT1 protein expression and lung function parameters was found.

Only a single study by Nakamuru et al. so far described decreased levels of SIRT1 activity in peripheral lung tissue from patients with COPD compared to nonsmoker and smoker healthy subjects. The authors also found that in the human monocytic cell line U937, hydrogen peroxide-induced oxidative burst led to a significant decrease of SIRT1 activity without changes in SIRT1 protein expression. Notably, more prolonged cell stimulation with hydrogen peroxide decreased the levels of SIRT1 protein expression as well as of SIRT1 activity [21].

Along with decreased SIRT1 activity levels, in the COPD patients, we found an increase of systemic oxidative stress index (TOS/TEAC ratio) compared to control groups. This finding further proves that an important characteristic of COPD is the augmented burden of oxidative stress as consequence of both increased oxidative status and decreased antioxidant defense. Importantly, in our study, while total oxidative status was similar between the COPD and the smokers, the total antioxidant capacity was the lowest in the COPD compared to both the healthy subjects; in fact, a significant correlation between the total antioxidant capacity and SIRT1 activity was found exclusively in the COPD patients.

The accumulation of ROS released from circulating leukocytes participating in chronic inflammatory response is among the mechanisms involved in COPD development and progression [28]. However, how much systemic inflammation is present in stable COPD and whether it can be relevant in the disease progression remains a controversial issue [11, 29]. Nonetheless, systemic manifestations of COPD along with clinical phenotypes and occurrence of exacerbations are aspects representing unresolved key questions in our understanding of COPD [30]. In this study, the positive correlations found in COPD patients of PBMC-derived SIRT1 activity with altered pulmonary function tests and plasmatic total antioxidant capacity support the occurrence of disease-specific systemic alterations at least in mechanisms of oxidative stress control.

The positive correlation existing between SIRT1 activity in PBMCs and parameters of airway obstruction and its severity (measured by FEV1/FVC and FEV1, resp.), as well as between SIRT1 activity and total antioxidant capacity in COPD patients, reinforces the importance of altered SIRT1 activity and SIRT1-mediated function as important mechanisms in COPD pathogenesis. These results also suggest that SIRT1 activity levels may be related to COPD disease per se beyond the presence of smoking habits and, importantly, point to a potential use of SIRT1 activity from PBMCs as an easily accessible biomarker for COPD.

Through its deacetylase activity, SIRT1 has been shown to regulate the expression of several molecules involved in restoring proper antioxidant and anti-inflammatory responses [19, 31]. Moreover, both natural and synthetic activators [32, 33] caloric restriction [34] and exercise training [19, 35] have been shown to enhance SIRT1 activity. Many previous studies have demonstrated the role of SIRT1 in contrasting oxidative stress by inducing the expression of antioxidant enzymes such as superoxide dismutase and catalase with clinically relevant consequences [36–38]. The correlation found in this study between SIRT1 activity and the total antioxidant capacity in COPD patients supports the effectiveness of antioxidant supplementation, which is now receiving a renewed interest as a useful therapeutic strategy for COPD. Among several compounds with antioxidant potential, N-acetyl-L-cysteine (NAC), a drug with both antioxidant and anti-inflammatory properties, has been recognized as a valid therapeutic agent for COPD, helpful to attenuate symptoms and prevent exacerbations [39].

Our finding of decreased levels of SIRT1 activity was obtained in a population of COPD patients in GOLD2 stage, suggesting the potential use of this parameter to identify COPD in its early stages. However, a limitation of this study is the absence of GOLD1 and the insufficient number of GOLD3 patients that did not allow us to thoroughly assess the value of SIRT1 activity as a prognostic biomarker. Studies including a COPD population with a larger spectrum of severity are therefore necessary to investigate whether SIRT1 activity could be used to stratify the patients with different GOLD stages.

## 5. Conclusions

Taken together, our findings indicate SIRT1 activity as a potential biomarker for COPD severity, easy to measure by being accessible through peripheral blood sampling, and potentially valuable to investigate as therapeutic target.

## Figures and Tables

**Table 1:** Main characteristics of the study population.

	HnS	HS	COPD	*p* value
Age, yr	66.05 ± 3.66	66.20 ± 4.98	68.58 ± 6.27	0.136
Sex ratio (M/F)	11/9	11/9	18/7	0.975
BMI, kg/m^2^	25.75 ± 2.54	23.01 ± 4.49	26.90 ± 5.99	0.071
FEV1, (% pred.)	110.10 ± 12.84	106.07 ± 11.24	53.96 ± 9.50^∗§^	<0.0001
FEV1/FVC, %	80.62 ± 5.02	80.92 ± 6.01	54.10 ± 7.88^∗^^§^	<0.0001
Pack/years	—	21.53 ± 12.08	52.34 ± 39.88	0.002

Data are expressed as mean ± SD. HnS: healthy nonsmokers; HS: healthy smokers; COPD: chronic obstructive pulmonary disease; BMI: body mass index; FEV1 (% pred.): forced expiratory volume in 1 second; FVC: forced vital capacity. ^∗^COPD versus HnS, *p* < 0.0001; ^§^COPD versus HS, *p* < 0.0001.

## Data Availability

The data used to support the findings of this study are available from the corresponding author upon request.
